# Voice Communication in Noisy Environments in a Smart House Using Hybrid LMS+ICA Algorithm

**DOI:** 10.3390/s20216022

**Published:** 2020-10-23

**Authors:** Radek Martinek, Jan Vanus, Jan Nedoma, Michael Fridrich, Jaroslav Frnda, Aleksandra Kawala-Sterniuk

**Affiliations:** 1 Department of Cybernetics and Biomedical Engineering, Faculty of Electrical Engineering and Computer Science, VSB-Technical University of Ostrava, 17. Listopadu 15, 708 33 Ostrava-Poruba, Czech Republic; jan.vanus@vsb.cz; 2 Department of Telecommunications, Faculty of Electrical Engineering and Computer Science, VSB-Technical University of Ostrava, 17. Listopadu 15, 708 33 Ostrava-Poruba, Czech Republic; jan.nedoma@vsb.cz (J.N.); michael.fridrich@vsb.cz (M.F.); 3 Department of Quantitative Methods and Economic Informatics, Faculty of Operation and Economics of Transport and Communications, University of Zilina, 01026 Zilina, Slovakia; jaroslav.frnda@fpedas.uniza.sk; 4 Faculty of Electrical Engineering, Opole University of Technology, Automatic Control and Informatics, 45-758 Opole, Poland; a.kawala-sterniuk@po.edu.pl

**Keywords:** automatic speech recognition, Smart Home (SH), LabVIEW, independent component analysis (ICA), least mean squares algorithm (LMS)

## Abstract

This publication describes an innovative approach to voice control of operational and technical functions in a real Smart Home (SH) environment, where, for voice control within SH, it is necessary to provide robust technological systems for building automation and for technology visualization, software for recognition of individual voice commands, and a robust system for additive noise canceling. The KNX technology for building automation is used and described in the article. The LabVIEW SW tool is used for visualization, data connectivity to the speech recognizer, connection to the sound card, and the actual mathematical calculations within additive noise canceling. For the actual recognition of commands, the SW tool for recognition within the Microsoft Windows OS is used. In the article, the least mean squares algorithm (LMS) and independent component analysis (ICA) are used for additive noise canceling from the speech signal measured in a real SH environment. Within the proposed experiments, the success rate of voice command recognition for different types of additive interference (television, vacuum cleaner, washing machine, dishwasher, and fan) in the real SH environment was compared. The recognition success rate was greater than 95% for the selected experiments.

## 1. Introduction

Spoken communication is the basic and most widely used way of transmitting information between people. The computer industry, where the goal is to make the computer a fully-fledged partner of human beings in spoken language, is no exception. This goal is pursued mainly because such a way of communication can be beneficial and can significantly facilitate a person’s life. Voice communication systems are increasingly used in industrial and social practice. In most applications, the usability is limited to a narrow area of tasks, i.e., dictionary limitations or predetermined commands which are to be recognized by a computer. Thus, various systems of machine and equipment control using voice commands or automatic dictation transcription are generally applicable. These systems are especially suitable in cases where a person’s eyes and hands are employed in other activities.

Dotihal et al. deals with the smart home (communication between the devices and gateway takes place through Power Line Communication (PLC) and the RF links either through TCP protocol or Message Queue Telemetry Transport (MQTT) protocol) with aims at controlling home appliances via smartphone and voice by using Alexa acting as a client [1]. Erol et al. built and tested a digital voice assistants system with an IoT device to control and simulate the process of assistive robotic workload within voice activation and control in order to improve human–robot interactions with IoT perspectives [2]. Social robotics is becoming a reality and voice-based human–robot interaction is essential for a successful human–robot collaborative symbiosis. The main objective of Diaz et al. is to assess the effect of visual servoing in the performance of a linear microphone array regarding distant ASR in a mobile, dynamic, and non-stationary robotic testbed that can be representative of real HRI scenarios [3]. Novoa et al. proposed to replace the classical black-box integration of automatic speech recognition technology in HRI applications with the incorporation of the HRI environment representation and modeling, and the robot and user states and contexts [4]. Grout in his paper, the role of the human-computer interface for remote, or online, laboratories are considered, for example, hand position/motion/gesture control and voice activation, which are modes of human-computer interaction (HCI) that are of increasing interest [5]. He et al. designed to implement an Arduino board alongside motion sensors and audio receiver to control a robot car by means of a cloud server, and IoT technologies, where the system for control the robot car by preset voice commands integrates Google Voice API [6]. Kennedy et al. investigate a new passive attack, referred to as voice command fingerprinting attack, on smart home speakers with experimental results on a real-world dataset suggest that voice command fingerprinting attacks can correctly infer 33.8% of voice commands by eavesdropping on encrypted traffic [7]. Knight et al. uses a combination of sensors, Raspberry Pis, camera feeds, and multiple interaction methods (voice, text, and visual dashboards) to facilitate laboratory communication for the fully interconnected laboratory of the future [8]. Kodali et al. presented a solution for applications, where is crucial for the development of Smart Cities as a whole along with Smart Homes (for example, switch a light source, HVAC systems, or any other electrical equipment on or off, by being physically present in the premises, remotely or automatically based on time or a sensor’s reading) with a speech recognition system to give the users a much more intuitive and natural mean to communicate with and control the connected devices [9]. Leroy et al. propose federated learning for keyword spotting to solve out-of-domain issues with continuously running embedded speech-based models such as wake word detectors with the aim of fostering further transparent research in the application of federated learning to speech data [10]. Based on the interactive experience principle of smart design in the smart building system Li et al. classifies and summarizes intelligent design from the “five senses” interaction, including visual interaction, voice interaction, tactile interaction, cognitive interaction, and emotional interaction, and proposes future research suggestions and directions and promotes the sustainable development of the smart building [11]. Liu designed and implemented of Smart Home Voice Control System based on Arduino [12]. Vanus used Voice communication within the monitoring of the daily living activities in smart home care [13] with the assessment of the quality of speech signal processing within voice control of operational –technical functions in the smart home [14].

This work is focused on the implementation of innovative voice control of operational and technical functions in the real Smart Home (SH) environment for subsequent testing of selected filtration methods. We tested and compared the methods of noise filtration by using an adaptative system (LMS) and a hybrid system (LMS+ICA). For this study’s purposes, the plug-and-play platform seemed to be the ideal tool for testing, or more precisely, connection with our virtual devices created in the LabVIEW graphically oriented interface. In this paper, we do not have ambitions to develop algorithms of recognition, but we present a way how to improve the effectiveness of speech recognizer via the mentioned adaptive systems. The partial goals of the work are as follows.

- To ensure control of operational and technical functions (blinds, lights, heating, cooling, and forced ventilation) in the SH rooms (living room, kitchen, dining room, and bedroom) using the KNX technology.
- To ensure recognition of individual commands for the control of operational and technical functions in SH.
- To record individual voice commands (“Light on”, “Light off”, “Turn on the washing machine”, “Turn off the washing machine”, “Dim up”, “Dim down”, “Turn on the vacuum cleaner”, “Turn off the vacuum cleaner”, “Turn on the dishwasher”, “Turn off the dishwasher”, “Fan on”, “Fan off”, “Turn on the TV”, “Turn off the TV”, “Blinds up”, “Blinds down”, “Blinds up left”, “Blinds up right”, “Blinds up middle”, “Blinds down left”, “Blinds down right”, “Blinds down left”, and “Blinds down middle”).
- To ensure data connectivity among the building automation technology, the sound card, and the speech recognition software tool.
- To upload sample additive interference in a real SH environment (TV, vacuum cleaner, washing machine, dishwasher, and fan).
- To ensure additive noise cancelling in the speech signal using the least mean squares algorithm (LMS) and the independent component analysis (ICA).
- To ensure visualization of the aforementioned processes of Visualization software application with a SH simulation floor plan; in this work, the measurement and processing of the speech signal were implemented using the LabView software tool together with a database of interference recordings.
- To ensure the highest possible recognition success rate of speech command in a real SH environment with additive noise.

## 2. Related Work

As part of the currently addressed issue of “smart home” automation, automatic or semi-automatic control and monitoring of household appliances and the operational technical functions, such as lights, blinds, heating, cooling or forced ventilation, is provided. Amrutha focuses upon different steps involved for speaker identification using MATLAB Programming with a speech recognition accuracy of more than 90% within Voice Controlled Smart Home [15]. Kamdar elaborates on the different methods of integrating voice recognition technology in home automation systems [16] Kango describes networked smart home appliances like a ubiquitous culture within SH [17]. Smart appliances often use innovative technologies and communication methods [18] that enable a variety of services for both the consumers and the manufacturers. Smart homes can, therefore, be defined as those that have the characteristics of central control of home devices, networking capabilities, interaction with the users via smart interfaces, etc. For natural interaction with the users, one of the most user-friendly methods is vocal interaction (VI), which corresponds to the physical environment of a smart home. System VI, which can be accessed from, for example, the garage, the bathroom, the bedroom, or the kitchen, requires a distributed set of microphones and speakers together with a centralized processing unit [19]. Automatic Speech Recognition (ASR) can be divided into three basic groups [20]. The first group consists of isolated word recognition systems (each word is spoken with a pause before and after the speech, for example in banking or airport telephone services). The second group comprises small glossary systems for application commands and control, and the last group consists of large glossary systems for continuous speech applications. From the ASR perspective, the smart home system is a mixture of the second and third group, wherein it is possible to dictate e-mail messages and use grammatically limited commands for household management, the so-called command and control vocabulary, etc. Predominantly, we can classify the ASR system by means of vocal interaction in two main categories: first, these are specific control applications that form the essence of smart homes (voice control of operational and technical functions and appliances), and, second, there are general vocal applications that can be used in all ASR systems [19]. Using a computer speech recognition technology, a multipurpose wireless system can be designed and created and such a system can turn off and on any household electrical appliance depending on the user’s voice command. Thoraya Obaid et al. [21] proposed a wireless voice system for the elderly and the disabled. The proposed system has two main components, namely a voice recognition system and a wireless system. The LabVIEW software was used to implement the voice recognition system. ZigBee modules were used for wireless communication. The proposed system only needs to be “trained” once. Based on the data received by and stored in the wireless receiver connected to the appliances, the required operations are performed. Another home automation system was designed by Dhawan S. Thakurand and Aditi Sharma [22]. The proposed system can be integrated as a standalone portable unit; allows wireless control of lights, fans, air conditioners, televisions, security cameras, electronic doors, computer systems, and audiovisual equipment; and can turn on or off all appliances that are connected to the electrical network. However, more complex commands can be managed through a set of alternatives where the vocabulary is limited. To handle these tasks, syntax, which specifies the given word and phrase with their admissible combinations (alternatives), is required [19]. Dictation involves automatic translation of speech into the written form. Dictation systems include large vocabulary and, in some cases, applications that include additional professional dictionaries for the application [23]. Domain-specific systems can thus increase recognition accuracy [19]. Many factors in the ASR system for VI can be regulated. For example, speech variability is generally of limited use. Language flexibility can be limited by a suitable grammatical proposal, etc. The ability to accurately recognize the speech captured that has been limited depends primarily on the vocabulary size and the signal-to-noise ratio (SNR). Thus, recognition can be improved, first, by reducing the size of the vocabulary and, second, by improving the signal-to-noise ratio. The size of vocabulary constraints in VI systems is based on grammar. Reducing vocabulary, for example by shortening individual commands, can lead to improved recognition [24,25]. Similarly, the quality of speech captured affects the accuracy of recognition [26]. Real-time response is another required characteristic. System performance is affected by three aspects: recognition speed, memory size requirements, and recognition accuracy. These aspects are in conflict with each other, as it is relatively easy to improve recognition speed while reducing memory requirements at the expense of reducing recognition accuracy [27]. The task of designing a voice recognition system is, therefore, to reduce the size of the vocabulary at each moment of the conversation as much as possible. ASR systems often use specific domains and specific applications tailored to improve performance, but vocabulary size is important in any general ASR language, regardless of the technique used in the implementation. Some systems have been designed from the ground up to examine the effects of vocabulary limitation, such as the Bellcore system [25], which contains up to 1.5 million individual words. Recognition accuracy decreased linearly with a logarithmic increase in directory size [25]. ASR systems currently are widely used also in the field of industrial robotics [28], in the field of wheelchair steered [29], in the field of defense and aviation [30], and in the field of telecommunications industry [31]. The IoT platform [32] within the cyber-physical system [33], which can be understood as a combination of physical [34], network [35] and computational processes [36,37], also plays an important role in current voice control applications. Speech contains information that is usually obtained by processing a speech signal captured by a microphone using sampling, quantization, coding [38], parametrization, preprocessing, segmentation, centring, pre-emphasis, and window weighting [39,40]. The next step is speech recognition with

- statistical approach for continuous speech recognition [41] with different approaches [42] for speech recognition system’s [43] using the perceptual linear prediction (PLP) of speech [44], for example,
  - Audio-to-Visual Conversion in Mpeg-4 [45],
  - acoustic modeling and feature extraction [46],
  - speech activity detectors [47] or joint training of hybrid neural networks for acoustic modeling in automatic speech recognition [48],
- the RASTA method (RelAtive SpecTrAl) [38], and
- the Mel-frequency cepstral analysis (MFCC), for example,
  - dimensionality reduction of a pathological voice quality assessment system [49],
  - content-based clinical depression detection in adolescents [50],
  - speech recognition in an intelligent wheelchair [51],
  - speech recognition by using the from speech signals of spoken words [52],
- the hidden Markov models (HMM) [53], and
- artificial neural networks (ANN) [54], for example,
  - feed-forward Neural Network (NN) with back propagation algorithm and a Radial Basis Functions Neural Networks [55],
  - an automatic speech recognition (ASR) based approach for speech therapy of aphasic patients [56],
  - fast adaptation of deep neural network based on discriminant codes for speech recognition [57],
  - implementation of dnn-hmm acoustic models for phoneme recognition [58],
  - combination of features in a hybrid HMM/MLP and a HMM/GMM speech recognition system [59], and
  - hybrid continuous speech recognition systems by HMM, MLP and SVM [60].

An essential part of speech signal processing is also the suppression of additive noise in the speech signal using single-channel or multichannel methods [61], for example, single-channel methods like

- speech enhancement using spectral subtraction-type algorithms [62],
- use of complex adaptive methods of signal processing [63,64],
- model-based speech enhancement [65,66],
- increasing additive noise removal in speech processing using spectral subtraction [67], and
- noise reduction of speech signal using wavelet transform with modified universal threshold [68] or denoising speech signals by wavelet transform [69].

Multichannel methods include

- the least mean square algorithm (LMS) [70,71],
- the recursive least squares algorithm (RLS) [72,73],
- the independent component analysis (ICA) [74,75], and
- the principal component analysis (PCA) [76,77] or beamformer (BF) methods for speech acquisition in noisy environments [78], to linearly constrained adaptive beamforming [79] with a robust algorithm [80].

## 3. The Hardware Equipment in SH

### 3.1. SH Automation with the KNX Technology

**Figure 1:**
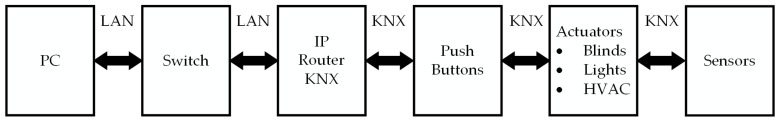
Block diagram of PC and KNX technology connection using an IP router.

Individual modules of the KNX technology were used for the implementation of SH automation. The KNX technology is designed for complex automation of intelligent buildings and households in accordance with European standard EN50090 (European Standard for Home and Building Systems) and ISO/IEC 14543 standard. It is used not only to control the shading elements (blinds, shutters, and awnings), but also to control the lighting (dimmable lights and lights being switched), the heating in the house, and, also, to control the other equipment in the building. It combines all technological parts in the house into one, logically arranged system, to increase the comfort of living. Based on the preference for building automation voice control by seniors, the elderly and the disabled, to support independent living, GHOST visualization software for voice control of operational and technical functions in SH was designed and tested. The GHOST visualization software connects the environment for recognition of individual voice commands and the KNX technologies [81]. The connection between the computer with voice control and the KNX communication bus, within the voice communication, was implemented using a Siemens KNX IP Router N146 module (5WG1 146-1AB01) (Figure 1). The module described has a KNX interface on one side and an Ethernet connector on the other side. However, when using this router, it is necessary to communicate over the network using the UDP protocol. Due to the automatic assignment of IP addresses, a switch was added between the computer and the KNX IP Router.

The next step is the software adaptation of the individual protocols (KNX and UDP). Input data in the form of component addresses and states of the individual modules can be changed using the ETS application. The method used is based on mapping IP addresses and UDP messages sent via the bus during each change in the sensor part of the system. KNX sensors, KNX bus buttons and switching, blind, dimming, and fan coil KNX actuators were used to control the operational and technical functions in the bedroom, kitchen, hallway, bathroom, and living room.

### 3.2. Steinberg UR44 Sound Card

In a classic USB mode, special drivers are required for Windows or Mac OS X, and the card then supports the ASIO, WDM, or Core Audio standards established (Table 1).

### 3.3. RHODE NT5 Measuring Microphones

The RHODE NT5 microphone is a small-diaphragm condenser microphone for recording sound sources, consisting of an externally deflected condenser, a 1/2” capsule with a gold-covered diaphragm, an active J-FET impedance transducer with a bipolar output buffer and dual power supply (Table 2).

## 4. The Software Equipment in SH

### 4.1. ETS5 - KNX Technology Parametrisation

**Figure 2:**
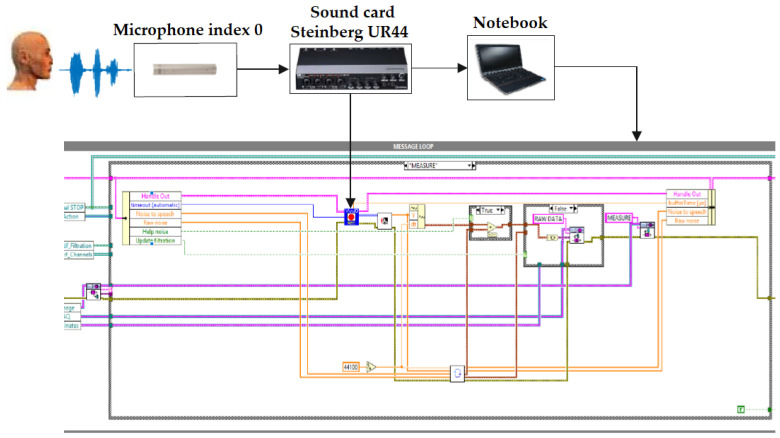
The main loop for continuous data reading.

**Figure 3:**
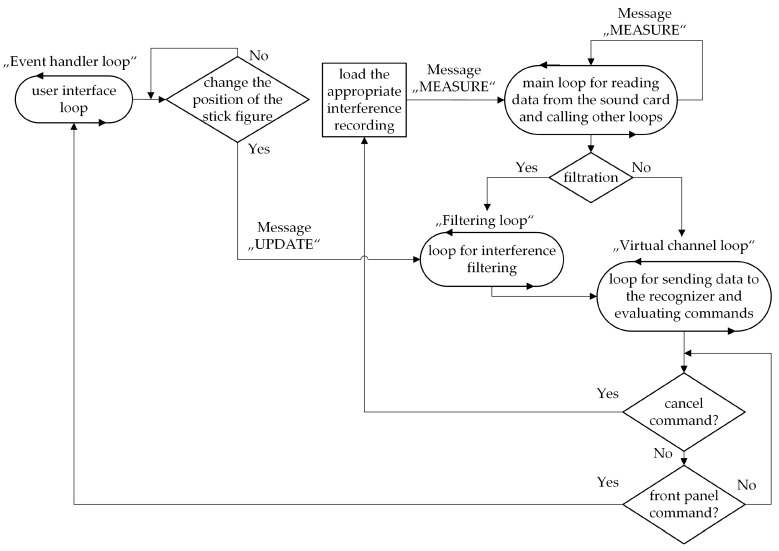
Simplified block diagram of the “smart home” algorithm.

The ETS5 SW tool was used for the actual parametrization of the individual modules (sensors, bus buttons, and actuators) of the KNX technology (Figure 2 and Figure 3).

### 4.2. LabVIEW Graphical Development Environment

The LabVIEW (Laboratory Virtual Instruments Engineering Workbench) graphical development environment is a product made by National Instruments, and this environment enables programming in a specific graphical programming language called “G”. This makes it intuitive even for inexperienced programmers/beginners, allowing programming without a deeper knowledge of syntax. The environment is, therefore, at the level of, for example, the C language, but unlike this language, it is not oriented towards the text, but graphically. The final product of this development environment is called a virtual instrument (Virtual Instrument, abbreviated VI), because its character and activity resemble a classic device in its physical form. Therefore, the virtual instrument is a basic unit of every application created in this development environment and contains [82] the following.

- Interactive Graphical User Interface (GUI)—the so-called front panel, which simulates the front panel of a physical device. It contains objects, such as controls and indicators, which can be used to control the running of the application, to enter parameters and to obtain information about the results processed.
- Block diagram, in which the sequence of evaluation of the individual program components (the program algorithm itself, their interconnection and parameters) is defined. Each component contains input and output connection points. The individual connection points can be connected to the elements on the panel using a wiring tool.
- Subordinate virtual instruments, the so-called subVI. The instrument has a modular, hierarchical structure. This means that it can be used separately, as an entire program, or as its individual subVI’s. Each VI includes its icon, which is represented in the block diagram, and a connector with locations connected for the input and output parameters.

The sequence of the program run is given by the data flow. The block diagram node is executed when it receives all the inputs required. After the node is executed, it creates the output data and passes the data to another node in the data stream path. The movement of data across the nodes determines the order of execution of the VI and the functions in the block diagram.

### 4.3. Speech Recognition 

A commercially available recognizer by Microsoft within the Windows OS was used for the actual speech recognition.

## 5. SW Application for Automation Voice Control in a Real SH

### 5.1. Visualization

One of the goals of the work is to create a model that will correspond to the recordings measured from a real SH environment using the LabView software. Based on this requirement, a visualization of a smart home was created. A commercially available Windows recognizer by Microsoft was used for voice control of the visualizations.

### 5.2. Speech Recognition

In order to be able to communicate with the recognizer, it is necessary to install the Speech SDK 5.1 driver, which works on the principle of converting a voice command into text. It is freely available software allowing developers to apply speech synthesis and recognition in Windows from various programming languages. A freely available VI (Speech recognition engine) was used for the communication between LabVIEW and the recognizer, which only needs to define the input field of the commands in a string format to the Grammar dictionary input connector, according to which the voice commands are compared. Then, the result of the command compared is converted into text at the output from the “Recognized command”.

### 5.3. Virtual Cable Connection

Therefore, in order to be able to filter voice commands to which interference was added and, thus, to send the data filtered to the recognizer, it was necessary to install the VB-CABLE (Virtual Audio Cable) program. It is software for transferring audio streams between applications or devices. It creates a set of virtual audio devices (so-called Virtual Cables), each of which is composed of a pair of input/output endpoints. Any application can then send an audio stream to the output part of the virtual cable, and another application can receive this stream through its input part. All transmissions are made digitally and there is no loss of quality. The program is suitable for recording audio output of the applications in real-time or for transferring the audio stream to another application, in which it is further processed.

On the computer, it is necessary to enable recording from this virtual connection in the audio settings and to disable other devices (microphone integrated in the laptop and the Steinberg UR44 sound card, or other devices).

### 5.4. The Main Loop for Data Reading

One microphone, which is set to index 0, was used for voice control of the visualizations. All visualizations contain a main loop, the task of which is to continuously read data, normalized it, add interference to speech and send this data to other loops. The measuring chain is shown in Figure 2.

As described above, the output data from the sound card is represented by a signed int32 resolution $\left(-231+231-1\right)$. Therefore, it is necessary to recalculate the amplitude values so that the input signals were first indexed and divided by the value of $\frac{2^{32}}{2}$.

If the state for loading interference recordings is activated, these interferences are added from the database to the real speech. As the interference recordings are approximately 5 s long, but the data collection from the sound card can take any time, it was necessary to synchronize the lengths of these signals. This is achieved using subVI rotation sums, when the time window of data collection is first detected and, based on this window, the time window of interference recordings is then defined. This is subsequently read and moved forward by another time window. As soon as you move to the end of the recording, the entire cycle starts from the beginning.

### 5.5. Visualization of a “Smart Home”

The application consists of a user interface loop, which is used to turn on/off the filtration and to capture the change in the position of the person icon, whose task is to simulate the part of the house in which the user is currently located. For example, if the person icon is placed in the bathroom area, it is not possible to activate lights or other devices located in other parts of the house (Figure 3).

If the position changes, the message “UPDATE” is triggered in the loop intended for filtering, where the data on the current coordinates of the occurrence of the simulated person (the person icon) is transferred, and the room the person is located in is evaluated. Subsequently, the data is converted into variables and stored in a cluster.

### 5.6. Glossary of Commands

The glossary for a “smart home” is defined by commands for switching the lights on/off and closing/opening the blinds, which can be used in all rooms. Other commands can then be used within the specific room (Table 3).

### 5.7. Application Control

**Figure 4:**
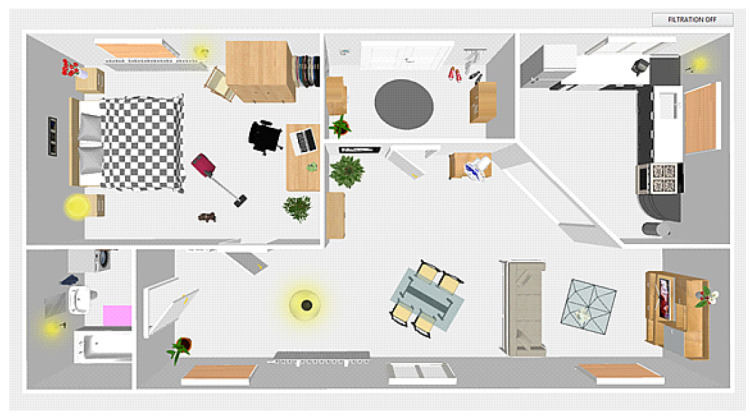
Front panel of the “smart home” application.

The “smart home” application consists of 5 rooms, namely, a bathroom, a bedroom, a hall, a living room, and a kitchen, in which voice commands can be issued. After turning on the application, the person icon is placed in the initial position, which is located in the hall. This state is the default, so it is possible to issue voice commands, but it is not possible to respond to them. For voice control, it is always necessary to place the person icon in a preselected position, where the command is executed based on the evaluation of the coordinates. For example, if the user wants to pull the blinds in the bedroom, they must move the person icon to this position, see Figure 4. This prevents unwanted conditions where the user moves the person icon into the kitchen, for example, and wants to control the device in other parts of the house.

Figure 4 shows the front panel of our virtual SMART Home. The whole house is functional and fully distributed with actual recordings from a real house. Within the recording process, several thousands of real recordings of the selected interference sources (television, vacuum cleaner, washing machine, dishwasher, and fan) were made. It is possible to place a “figurine” into the picture that simulates a user controlling the household via voice. “Noises” coming from the individual sources (household appliances) were recorded from various distances and positions. The retention measurements were taken in a semi-reflective room when the background noise level was being changed during the recording via the user’s movement within the room. According to the scenario, every command was repeated 100 times for the individual position, and 20 different speakers participated (10 men and 10 women, various age). These experimental scenarios bring us results in the form of a ratio. The aim was to provide a realistic view of the importance of filtering in commercial speech recognizers in a real environment. Within the virtual device, the user’s movement in the room was being simulated, and the level of background noise was changing. In general, the interference level ranged between 0 and 20 dB. Our attempt was to come as close as possible to real scenarios. In other words, we wanted to create a virtual device that would provide additionally checked records for the purposes of development and testing of the filtration methods.

## 6. The Mathematical Methods Used

### 6.1. Least Mean Squares Algorithm

The LMS algorithm is one of the most widespread and most widely used adaptive algorithms employed in current practice. The strength of the LMS algorithm lies in its simplicity and mathematical incomplexity [71]. These algorithms are based on a gradient search algorithm, also called the maximum gradient method. The dependence of the adaptive FIR filter output error signal standard deviation on the filter coefficients is a quadratic curve with one global minimum [72]. The output equation is defined according to Equation (1).

(1) y(n)=w(n)x(n),

(2) $$y\left(n\right)=w^{T}\left(n\right)x\left(n\right).$$

Filter recursion is

(3) w(n+1)=w+2μe(n)x(n),

where μ represents the step size of the adaptive filter, **w**(*n*) is a vector of filter coefficients, and **x**(*n*) is the input vector of the filter.

**Figure 5:**
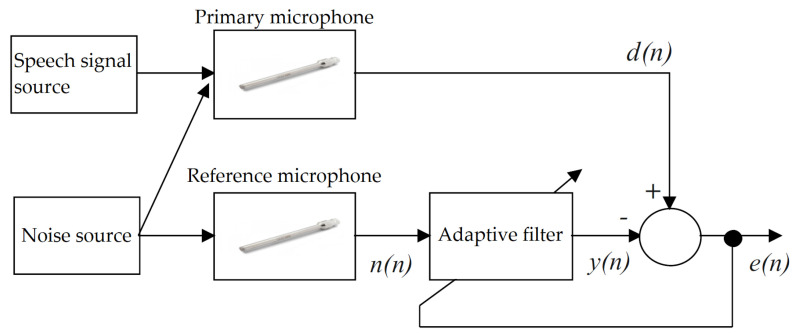
General block diagram of an adaptive system.

Figure 5 shows a general diagram of an adaptive filter system where *y*(*n*) represents the output signal of the filter, *d*(*n*) represents the noisy signal measured, *n*(*n*) represents the noise from the reference sensor and *e*(*n*) represents the deviation of the output signal from the measured one.

### 6.2. Independent Component Analysis

Independent component analysis is one of the blind source separation methods (BSS), which are methods used to estimate independent sources from multichannel signals. BSS methods in the field of digital signal processing consist in a situation where several signals are mixed together, and the task is to find out what the source signals looked like.

Independent component analysis, as one of the possible solutions to the “cocktail-party problem”, is a statistical and computational method for detecting hidden factors that are the basis of groups of random variables, measurements, or signals. This method defines a model for observing many randomly variable data, which is typically defined as a large sample database. In this model, the data variables are considered as linear mixtures of some unknown hidden variables, and the mixing system is not known. Hidden variables are considered non-Gaussian and independent of each other and are called independent components of the data observed. These independent components, also called sources or factors, can be found using the ICA method. Independent component analysis is superficially related to principal component analysis and factor analysis. However, ICA is a much more powerful method capable of finding the underlying factors or resources, even if these other methods fail completely. There is the following transformation,

(4) x(k)=As(k)+v(k),

where **A** represents a mixing matrix. The goal is to find the separation matrix, i.e., a matrix **H** having a size of *N*∗*M* to which the following applies, $H=H^{-1}A$. The two basic limitations of the ICA method include the impossibility to recover the energy of source signals and the impossibility to maintain the order of the source signals. Thus, the output components have a different amplitude with respect to the input signals, and when the ICA method is applied again, the components have a different order and polarity of signals.

These limitations are compensated by multiplying the resulting separation matrix **H** by two matrices. Matrix **P** is a permutation matrix that adjusts the order of the separated components, and matrix **D** is a diagonal matrix that adjusts the energies of separated signals. In summary, therefore, the following applies:

(5) $$H=A^{-1}\mathrm{DP}.$$

### 6.3. Prerequisites for ICA Method Processing

**Figure 6:**
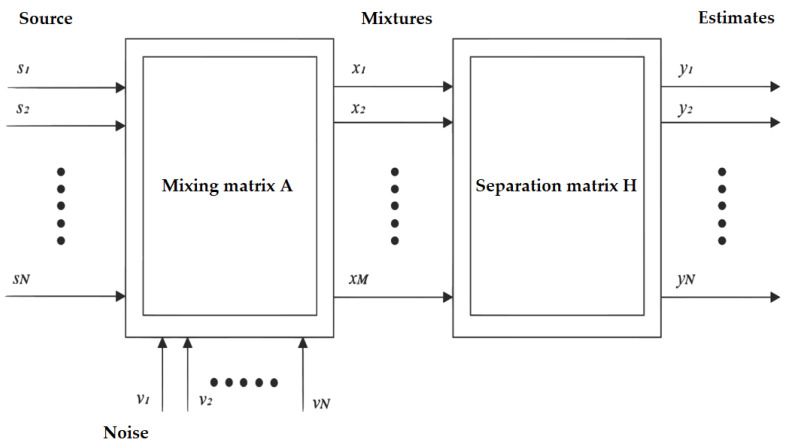
Basic model of the independent component analysis (ICA) method.

Before the actual application of the ICA method (see Figure 6), preprocessing in the form of centering and bleaching the input signals is performed [76]. The centering is supposed to remove the DC component from the signal edited. In this step, the signal’s mean value is subtracted from the input signal. Therefore, the following applies.

(6) $$x_{c}\left(k\right)=x\left(k\right)-\frac{1}{K}\sum_{1=k}^{K}x\left(k\right)$$

After processing, the inverse process can be performed using a separation matrix **H** and estimates *y*(*k*):

(7) $$y_{c}\left(k\right)=y\left(k\right)-H\frac{1}{K}\sum_{1=k}^{K}x\left(k\right)$$

Bleaching is a process of modifying a signal after the application of which the input signals are uncorrelated and are scattered per unit. Therefore, if the sensor signals *x*(*k*) are bleached, then their correlation matrix is equal to the unit matrix: $E\{xx^{T}\}=I$ =. This transformation can be written as follows,

(8) $$x_{B}\left(k\right)=\mathrm{Bx}\left(k\right),$$

where **Bx**(*k*) denotes a bleached vector and **B** represents the so-called whitening dimension matrix *N*∗*M* for which the following applies: ${\mathrm{BB}}^{T}=I$. The Singular Value Decomposition (SVD) method can be used to calculate the bleach matrix and to design a bleach matrix using eigenvectors and eigenvalues of the correlation matrix of mixture vectors.

## 7. Experimental Part—Results

### 7.1. Selected Filtering Methods and Recognition Success Rate

To suppress interference, the ICA method was selected together with the adaptive method with the LMS algorithm. Despite its simplicity and mathematical incomplexity, the LMS algorithm produced good-quality results of the global SNR.

### 7.2. Search for Optimal Parameter Settings for the LMS Algorithm

**Figure 7:**
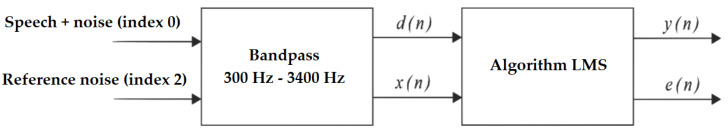
Filtration measuring chain for the LMS algorithm.

As, in the visualizations, it is not possible to determine, in advance, what the next command will be (for this reason the ideal parameters of the LMS algorithm cannot be set), it was first necessary to perform offline identification. This was performed by finding the optimal values for each command and interference according to the global SNR. From these values found, the best filter length M was then selected as well as convergence constant μ. The filtration takes place in two steps (Figure 7), where the speech signal contaminated with interference and the reference noise are first fed to a bandpass filter set at 300 Hz–3400 Hz, which is approximate frequency range corresponding to human speech. Then, the filtered signals are sent to the LMS algorithm, where *y*(*n*) is the filtered signal, and *e*(*n*) is the filtration error.

Table 4 shows that, with increasing interference energy, there will be greater demands on the adaptive filter. It means higher filter length M and convergence constant μ. When testing, the filter appeared to require a higher filtration length with increasing interference energy, but there is a problem when, at high values (filter length of 1.000 and higher), the useful signal, which is partially filtered, is distorted, and the filtration error increases. The same applies to the convergence constant, where, at high values (above 0.1), the scales get disbalanced and the filter thus becomes unstable. Computing time is another problem. The greater the length of the filter and the smaller the convergence constant, the longer the calculation will take and vice versa. This creates a conflicting situation where the effort is for the best possible filtration in a minimum of time.

### 7.3. Independent Component Analysis

**Figure 8:**
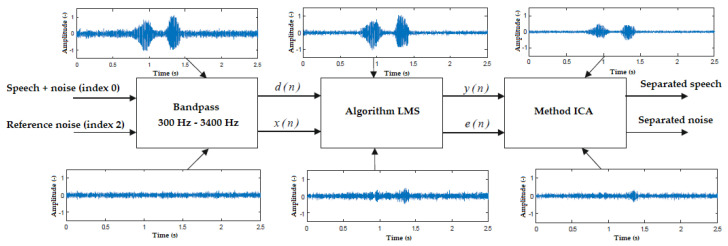
Measuring chain of hybrid LMS and ICA filtrations.

Only one microphone was used for voice control of the visualizations and, therefore, it is not possible to solve the classic “cocktail party problem”. For this reason, hybrid filtration was used (Figure 8), where the ICA method was implemented behind the output of the adaptive filter (Table 5). After passing through the bandpass, the signals are sent to the LMS algorithm and filtered out. It is clear from the waveforms that the algorithm significantly suppresses the interference, but, at the same time, the filtration error increases. This is due to the effort of the LMS algorithm to suppress the interference as much as possible while partially filtering the speech. This is one of the features of adaptive algorithms that must be taken into account. In LabVIEW, a function in the Signal Processing → Time Series Analysis library was used.

### 7.4. Recognition Success Rate

One-hundred iterations were performed for each command, and, based on the recognized/unrecognized status, the recognition success rate was evaluated. The commands were spoken into the microphone at a constant distance of 15 cm. The recognizer had the lowest recognition rate for words ending in “off” (“light off”, “i-stop off”, and “radio off”). This can be caused by the phonetic aspect of the command, which has low energy. Another reason is the property of adaptive filters when the word is suppressed (slightly filtered).

## 8. Discussion

Three commands were tested for washing machine interference: “Light on”, “Light off”, and “Turn off the washing machine”. Table 6 shows that the success rates before filtration were 28%, 21%, and 85%, respectively. When the washing machine is switched off, the high success rate of the filtration is caused mainly by the fact that the recognizer itself has a learning algorithm, wherein it returned previous values, which can be seen in the filtration results, where the success rate of the recognition was worse.

For vacuum cleaner interference (Table 7), the average success rate before filtration was only 1%, when only the commands “dim up” (5%) and “dim down” (3%) were recognized. After filtration, the average success rate for the LMS algorithm was 80% and the average success rate for the ICA algorithm was 86%. The lowest success rate was recorded for the “light off” command, where the filtration for the algorithm was only 27%, and 45% for the ICA method.

For fan interference (Table 8), the average success rate before filtration was only 11%, when the commands “light on” (42%) and “light off” (24%) were most recognized. After filtration, the average success rate for the LMS algorithm was 82% and the average success rate for the ICA algorithm was 91%. The lowest success rate was recorded for the “fan off” command again, where the filtration for the algorithm was only 18%, and 13% for the ICA method.

For dishwasher interference (Table 9), the average success rate before filtration was only 2%, when the command “Turn off the dishwasher” was most recognizable, but, on the other hand, it had zero success rate after filtration. The average success rate for the LMS algorithm and the ICA method was the same, namely, 85%.

**Figure 9:**
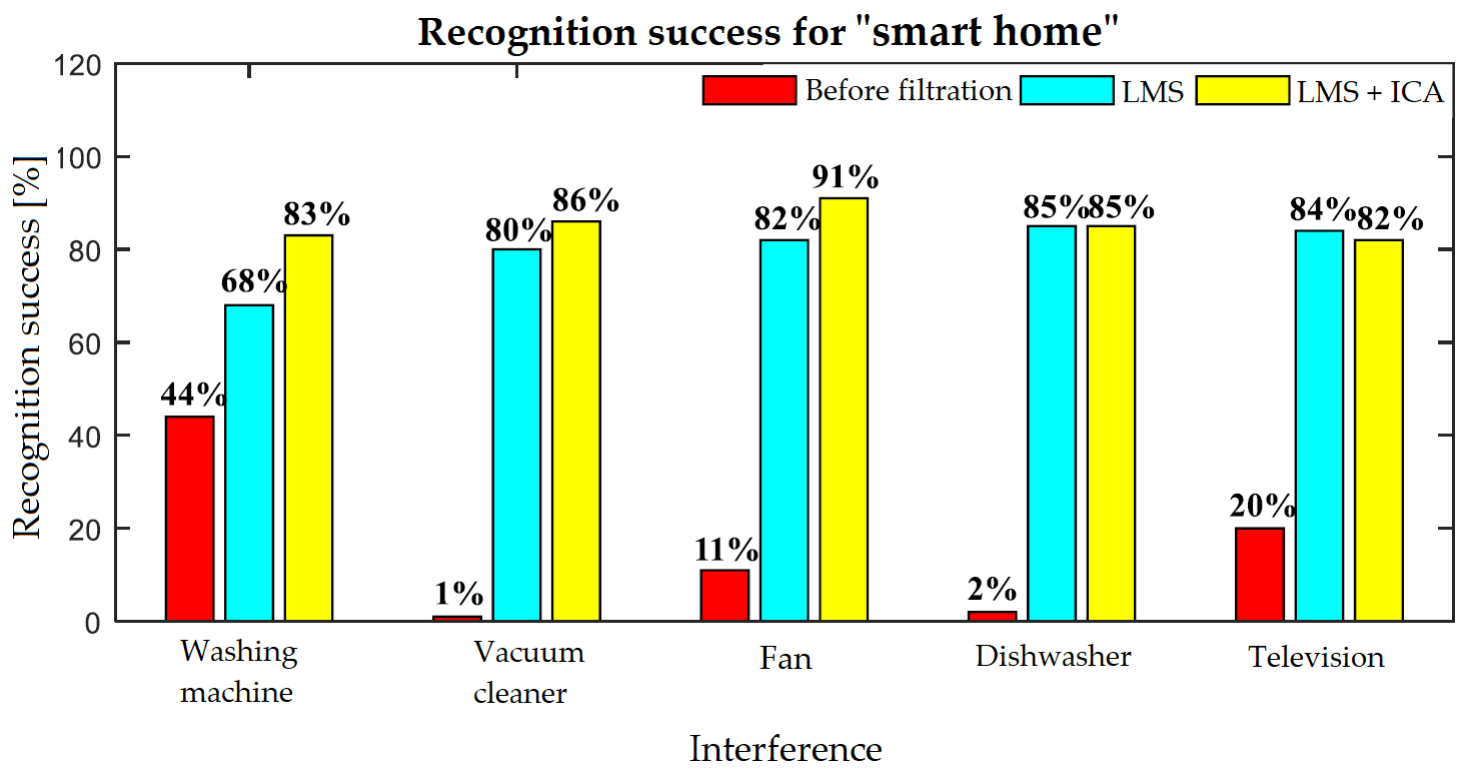
Recognition results for speech commands for additive interference in the real SH environment.

With the TV on (Table 10), the average success rate before filtration was only 20%, wherein the commands “blinds up middle” (42%), “blinds down left” (24%), “blinds down right” (21%), and “blinds down middle” (20%) were most recognizable. This is due to the fact that the recognizer was able to capture such long words between pauses of dialogues from the television. The average success rate for the LMS algorithm was 84% while, for the ICA method, it was 82%. The lowest recognition success rate after filtration was with the “blinds down” command, where the recognizer usually evaluated another alternative (“blinds down middle”, “blinds down right”, and “blinds down left”) (Figure 9).

**Figure 10:**
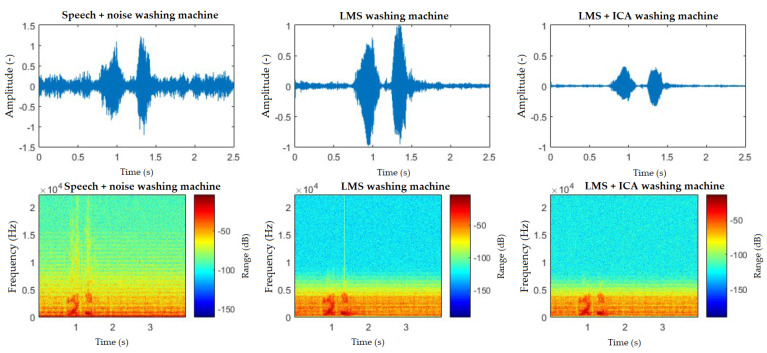
Comparison of spectrograms with closed windows, “light on” command, washing machine interference.

**Figure 11:**
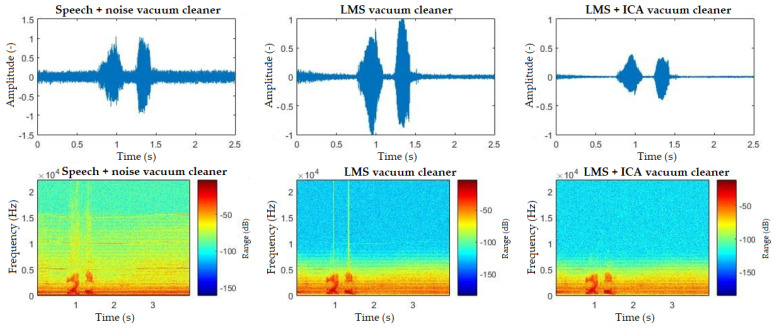
Comparison of spectrograms with closed windows, “light on” command, vacuum cleaner interference.

**Figure 12:**
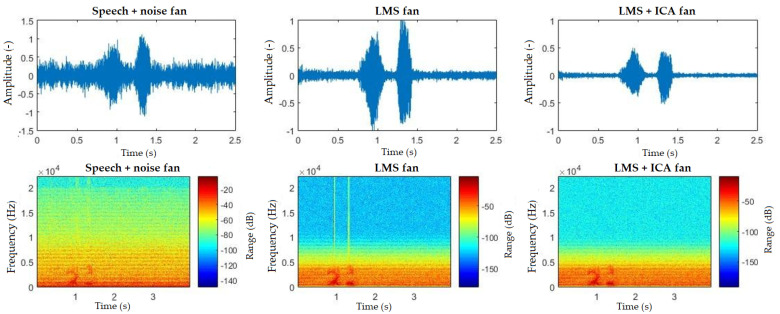
Comparison of spectrograms with closed windows, “light on” command, fan interference.

**Figure 13:**
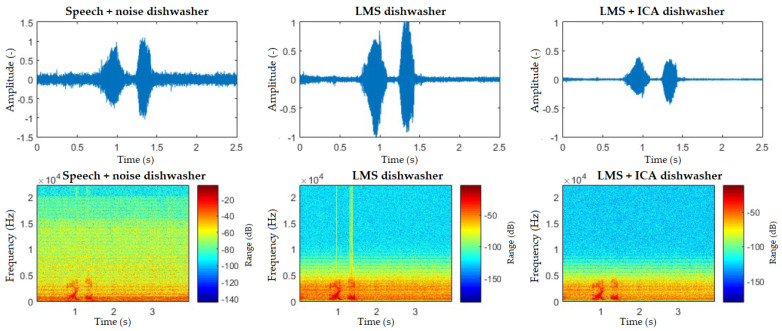
Comparison of spectrograms with closed windows, “light on” command, dishwasher interference.

**Figure 14:**
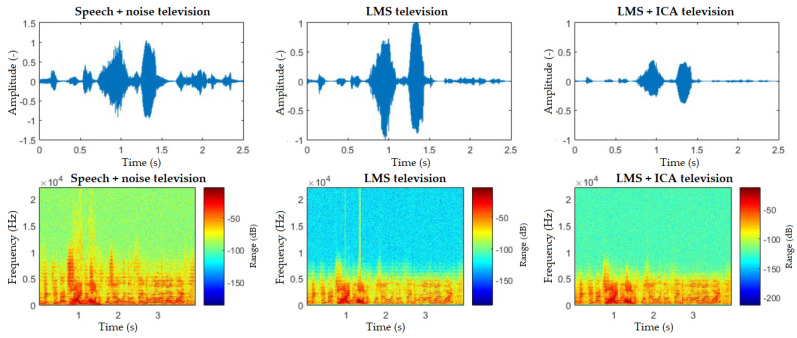
Comparison of spectrograms with closed windows, “light on” command, television interference.

The spectrograms (Figure 10, Figure 11, Figure 12, Figure 13 and Figure 14) show that the behavior of the appliances is similar after filtration, especially as for the washing machines and the vacuum cleaner. For the fan, the results are identical. This is mainly due to the uniform distribution of the noise, which is close to the Gaussian one. Furthermore, by theory, adaptive filters do not function well with these noises, and, as for the ICA method, this is a basic limitation when these interferences cannot be handled well. The spectrogram also shows that the best filtered interference was in the dishwasher. In the case of television interference, it can be seen, on the other hand, that the quality of filtration favors the LMS algorithm. The main reason is that only one microphone is used, so the classic principle of the ICA method cannot be addressed.

## 9. Conclusions

This study was focused on an innovative method of processing speech signals used for voice control of operational and technical functions in Smart Home, with subsequent testing of selected filtering methods. To control the operational and technical functions (blinds, lights, heating, cooling, and forced ventilation) in the SH rooms (living room, kitchen, dining room, and bedroom), a program for controlling the KNX technology was created using the ETS 5 software tool. A Microsoft recognizer was used to recognize the individual voice commands. To ensure visualization and data connectivity among the building automation technology, the sound card, and the SW tool for speech recognition, a LabView SW tool was used in this work together with a database of additive interference recordings in a real SH environment (television, vacuum cleaner, washing machine, dishwasher, and fan). A linear adaptive LMS filter and the ICA method were chosen to filter speech signals that contained additive noise from the real SH environment. The criterion for successful recognition was represented by a sequence of one hundred repetitions for each command based on which the recognized/unrecognized state was evaluated. During testing, commands for five types of interference were tested. The results show that the hybrid method showed a higher recognition success rate than the LMS algorithm, on average by 6%. The average recognition success rate before and after filtering was 64.2% higher for the LMS algorithm and 69.8% for hybrid filtering. The overall results reveal that hybrid filtration showed a higher success rate by only about 5%. Due to the computational complexity of the ICA method, it is much more advantageous to implement the LMS algorithm, which is capable of high levels of filtering despite its simplicity, but, with the increasing performance and quality of computer technology, there is room for more complex algorithms to address large tasks at relatively low cost.

In the next work, the authors will focus on optimizing the control of the operational and technical functions in SH and increasing the recognition success rate of the individual speech commands using appropriate speech recognition algorithms and appropriate algorithms for additive noise canceling in real time.

## Figures and Tables

**Table 1:** Steinberg UR44 sound card specifications.

Sound Card Type	USB
Number of analogue outputs	6
Number of microphone inputs	4
Number inputs	4
Number outputs	4
MIDI	YES
Phantom power supply	+48VDC
Sampling frequency	44.1 kHz, 48 kHz, 88.2 kHz, 96 kHz, 176.4 kHz, 192 kHz
Resolution	up to 24 bits at a maximum sampling rate

**Table 2:** RHODE NT5 microphone specifications.

Acoustic Principle	Pressure Gradient
Sound pressure level	143 dB
Active electronics	J-FET impedance converter with a bipolar output buffer
Directional characteristics	Cardioid (kidney)
Frequency range	20 Hz–20 KHz
Output impedance	100 Ω
Power supply options	24VDC or 48VDC
Sensitivity	−38 dB re 1 Volt/Pascal (12 mV @ 94 dB SPL) +/− 2
Equivalent noise level	16dBA
Output	XLR
Weight	101 g

**Table 3:** Glossary of commands for voice control of “smart” household.

Command	Room
“Light on”	All
“Light off”	All
“Turn on the washing machine”	bathroom
“Turn off the washing machine”	bathroom
“Dim up”	bedroom
Dim down	bedroom
“Turn on the vacuum cleaner”	bedroom
“Turn off the vacuum cleaner”	bedroom
“Turn on the dishwasher”	kitchen
“Turn off the dishwasher”	kitchen
“Fan on”	Hall
“Fan off”	Hall
Turn on the TV	Living room
Turn off the TV	Living room
“Blinds up”	Kitchen/hall/living room/bedroom
“Blinds down”	Kitchen/hall/living room/bedroom
“Blinds up left”	Kitchen/hall/living room
“Blinds up right”	Kitchen/hall/living room
“Blinds up middle”	Kitchen/hall/living room
“Blinds down left”	Kitchen/hall/living room
“Blinds down right”	Kitchen/hall/living room
“Blinds down left”	Kitchen/hall/living room
“Blinds down middle”	Kitchen/hall/living room

**Table 4:** Optimal parameter settings for the LMS algorithm, visualization of a “smart home”.

Interference	Filter Length M	Convergence Constant μ [-]
Washing machine	240	0.01
Vacuum cleaner	80	0.001
Fan	210	0.01
Dishwasher	40	0.01
TV	110	0.01

**Table 5:** ICA function block parameter settings.

Parameter	Value
Method	FastICA
Number of components	2
Number of iterations	1000
Convergence tolerance	0.000001

**Table 6:** The results of recognition success rate for washing machine interference.

LMS and ICA	Washing Machine Maximum Volume		
Command	Before [%]	LMS [%]	LMS + ICA [%]
“Light on”	28	100	100
“Light off”	21	45	70
“Turn off the washing machine”	85	60	78

**Table 7:** The results of recognition success rate for vacuum cleaner interference.

LMS and ICA	Vacuum Cleaner Maximum Volume		
Command	Before [%]	LMS [%]	LMS + ICA [%]
“light on”	0	100	100
“light off”	0	27	45
“blinds down”	0	95	100
“blinds up”	0	93	100
“Dim up”	5	100	100
“dim down”	3	100	100
“Turn off the vacuum cleaner”	0	45	60

**Table 8:** The results of recognition success rate for fan interference.

LMS and ICA	Fan Maximum Volume		
Command	Before [%]	LMS [%]	LMS + ICA [%]
“light on”	42	100	100
“light off”	24	28	90
“blinds down”	2	100	100
“blinds up”	5	91	100
“blinds down left”	3	88	100
“blinds down right”	0	83	100
“blinds down middle”	0	100	100
“blinds up left”	6	95	100
““blinds up right”	9	100	100
“blinds up middle”	15	100	100
“Fan off”	18	18	13

**Table 9:** The results of recognition success rate for dishwasher interference.

LMS and ICA	Dishwasher Maximum Volume		
Command	Before [%]	LMS [%]	LMS + ICA [%]
“light on”	0	100	100
“light off”	0	65	66
“blinds down”	0	100	100
“blinds up”	0	100	100
“blinds down left”	0	100	100
“blinds down right”	3	100	100
“blinds up left”	5	100	100
“blinds up right”	0	100	100
“Turn off the dishwasher”	10	0	0

**Table 10:** The results of recognition success rate for vacuum cleaner interference.

LMS and ICA	TV Maximum Volume		
Command	Before [%]	LMS [%]	LMS + ICA [%]
“light on”	60	100	100
“light off”	0	74	68
“blinds down”	0	62	51
“blinds up”	0	74	60
“blinds down left”	24	97	80
“blinds down right”	21	98	96
“blinds down middle”	20	100	98
“blinds up left”	15	91	88
“blinds up right”	8	74	90
“blinds up middle”	42	100	98
“Turn off the TV”	27	52	69
